# Atlanta Medical College

**Published:** 1860-10

**Authors:** 


### EDITORIAL AND MISCELLANEOUS.

### Atlanta Medical College.

The labors of the Sixth Course of Lectures in the above Institution terminated with the Annual Commencement, on the first day of September. We are gratified in knowing that perfect harmony—so essential to a successful course of instruction—existed between members of the class, and between teacher and pupil.

The commencement exercises were introduced at 10½ o'clock with prayer by Rev. J. S. Wilson, after which the Degree of Doctor of Medicine was conferred on sixty applicants, all of whom—with three or four exceptions—were present, and received the Diploma of the College. After the conferment of the Degree, Dr. Means reported the awards of Premiums, which had been made by committees appointed by the several members of the Faculty, offering premiums for the best Thesis on subjects connected with their respective branches, as follows:

To D. T. Richardson of Louisiania, for the best Thesis on subjects connected with Anatomy, *A Gold Lancet Case.* To G. L. Hunter, of South Carolina, for the best Thesis on subjects connected with Surgery, *A Gold Medal.* To J. M. Rendleman of Georgia, for the best Thesis on subjects connected with Physiology, a complete set of the "*Cyclopœdia of Medicine.*" To J. M. Collier of Georgia, for the best Thesis on subjects connected with Materia Medica, *A Pocket Case of Gilt Instruments.*

In the programme of exercises, then followed the Valedictory addresses on behalf of the Faculty and the Class. The Farewell of the Class, which will be found in the present number of the Journal, was pronounced by S. T. Beasley of LaGrange, Ga. It is short, but replete with rich sentiment, and was effectively delivered. Then followed that on the part of the Faculty, by Prof. A. Means, which was characterized by his usual richness of thought, and happy style of delivery. We regret exceedingly our misfortune, and that of our readers, in not being able to meet with the Doctor at the proper time to procure a copy for publication, in this number of the Journal. We hope to do so in time for our next issue. The exercises were witnessed by a very large, *interesting*, and attentive auditory.

On this interesting occasion, every face wore a cheerful smile, and all hearts were glad. Nothing was there to mar the pleasure of the occasion, save the thought that soon those so long and pleasantly connected must part.

And now, though the sweets of rest temptingly invite us from the arduous labors and weighty responsibilities connected with the duties of the Session, the rupture of agreeable and well-formed associations—with many never again to be restored—detracts much from the anticipated enjoyment.

Never, perhaps, did there assemble a Class of Medical Students more zealously devoted to their studies, and none during the term of one course have likely made greater proficiency. We delight to forward the interests of men, whose devotion to science gives indubitable evidence of their fitness for the noble, philanthropic and honorable profession of medicine.

The exercises of the Preparatory or Winter Course in this Institution, the advertisement of which was crowded out of our last number of the Journal, will open as usual on the first Monday in November next. The Faculty are satisfied, from their observation of its benefits to the student, for the past two winters, that it is highly important in fixing permanently in the mind, a knowledge of the fundamental principles of the Medical Sciences.

The Catalogue of Matriculates and Graduates of the Regular Course just closed, with Annual Announcement of the Regular Course for 1861, was expected to accompany this number of the Journal, but has been unavoidably delayed. It will be published by the tenth of October, instant.

